# Clinicopathologic significance of HIF-1α, p53, and VEGF expression and preoperative serum VEGF level in gastric cancer

**DOI:** 10.1186/1471-2407-8-123

**Published:** 2008-05-01

**Authors:** Sung Yong Oh, Hyuk-Chan Kwon, Sung-Hyun Kim, Jin Seok Jang, Min Chan Kim, Kyeong Hee Kim, Jin-Yeong Han, Chung Ock Kim, Su-Jin Kim, Jin-sook Jeong, Hyo-Jin Kim

**Affiliations:** 1 Department of Internal Medicine, Dong-A University College of Medicine, Busan, Republic of Korea; 2 Department of Surgery, Dong-A University College of Medicine, Busan, Republic of Korea; 3 Department of Laboratory Medicine, Dong-A University College of Medicine, Busan, Republic of Korea; 4 Department of Pathology, Dong-A University College of Medicine, Busan, Republic of Korea

## Abstract

**Background:**

Hypoxia influences tumor growth by inducing angiogenesis and genetic alterations. Hypoxia-inducible factor 1α (HIF-1α), p53, and vascular endothelial growth factor (VEGF) are all important factors in the mechanisms inherent to tumor progression. In this work, we have investigated the clinicopathologic significance of HIF-1α, p53, and VEGF expression and preoperative serum VEGF (sVEGF) level in gastric cancer.

We immunohistochemically assessed the HIF-1α, p53, and VEGF expression patterns in 114 specimens of gastric cancer. Additionally, we determined the levels of preoperative serum VEGF (sVEGF).

**Results:**

The positive rates of p53 and HIF-1α (diffuse, deep, intravascular pattern) were 38.6% and 15.8%, respectively. The VEGF overexpression rate was 57.9%. p53 and HIF-1α were correlated positively with the depth of invasion (*P *= 0.015, *P *= 0.001, respectively). Preoperative sVEGF and p53 levels were correlated significantly with lymph node involvement (*P *= 0.010, *P *= 0.040, respectively). VEGF overexpression was more frequently observed in the old age group (≥ 60 years old) and the intestinal type (*P *= 0.013, *P *= 0.014, respectively). However, correlations between preoperative sVEGF level and tissue HIF-1α, VEGF, and p53 were not observed. The median follow-up duration after operation was 24.5 months. HIF-1α was observed to be a poor prognostic factor of disease recurrence or progression (*P *= 0.002).

**Conclusion:**

p53, HIF-1α and preoperative sVEGF might be markers of depth of invasion or lymph node involvement. HIF-1α expression was a poor prognostic factor of disease recurrence or progression in patients with gastric cancers.

## Background

In gastric cancer, lymph node (LN) metastasis and distant metastasis have been established as the standard prognostic factors for relapse-free or overall survival. Angiogenesis is a crucial mediator of tumor progression. As tumors expand, diffusion distances from the existing vascular supply increase, resulting in hypoxia [1]. Hypoxia limits tumor growth, and tumors with poor vascularization fail to grow and form metastases [2]. However, hypoxia also affects tumor growth positively, by inducing cellular adaptations as well as angiogenesis and genetic alterations [3].

Hypoxia-inducible factor-1α (HIF-1α) is a transcription factor for many genes recognized to control the delivery of oxygen and nutrients via the induction of angiogenesis and glycolysis [4]. HIF-1α activates the transcription of vascular endothelial growth factor (VEGF), a key factor in tumor angiogenesis, and the expression of glucose transporters, glycolytic enzymes, and growth factors, which may promote tumor cell survival under hypoxic conditions [5].

Hypoxia has also been reported to induce wild-type p53 via a different pathway than DNA-damaging agents [6]. The hypoxic/anoxic induction of p53 selects for tumor cells that lack functional p53, and hence evidence diminished apoptotic potential [7]. The interaction of HIF-1α and p53, which affects tumor growth and clinical outcome, remains obscure to some extent. The overexpression of the HIF-1α and p53 proteins has been demonstrated in a variety of human cancers via immunohistochemistry (IHC) [8].

However, in gastric cancer, correlations of p53, HIF-1α and VEGF expression with preoperative serum VEGF (sVEGF) level with regard to clinicopathologic significance and prognosis are currently a matter of debate. In this study, we have examined, via IHC, the expression of HIF-1α, VEGF, and p53 proteins in surgical gastric cancer specimens. Also, we have examined preoperative sVEGF level for assessing the cliniclpathological significance and co-relation of each factor.

## Methods

### Patients and specimens

Paraffin-embedded tumor specimens from 114 patients, representing patients who had undergone curative gastrectomies at the Dong-A University Hospital and consecutively affiliated hospitals from March 2003, were utilized to evaluate the clinical significance of protein expression. None of the patients had received any preoperative treatments. The study was approved by the ethics committee (Dong-A Medical Center Institutional Review Board) and informed written consent was obtained from all patients before study entry.

### Blood samples and assays of sVEGF level

Peripheral venous blood samples were obtained within 7 days prior to gastrectomy, collected in plain tubes, permitted to clot, and were centrifuged at 3,000 rpm for 10 min at 4°C within one hour of collection in order to obtain the serum. The serum was aliquoted and stored at -270°C until being assayed. The VEGF levels from the sera of patients and the standard solutions supplied were measured with a commercially available system (Quantikine hVEGF Immunoassay, R&D Systems, USA) in accordance with the manufacturer's instructions. All serum samples were measured by an investigator who was blinded to the clinical data.

### Immunohistochemistry

Paraffin-embedded tissues were cut into 5-μm-thick sections and subjected to immunohistochemical analyses conducted via the avidin-biotin-peroxidase complex method.

#### HIF-1α, p53, and VEGF

The primary antibody for HIF-1α protein used in this study was monoclonal IgG 2b (clone H1α 67) (1:50; Novus Biologicals, Littleton, CO, USA). The primary antibodies used for VEGF proteins were rabbit polyclonal Ab A-20 (1:100; Santa Cruz Biotechnology, Santa Cruz, CA, USA) and p53 protein mouse monoclonal antibody (1:100; Dako, Glostrup, Denmark). After incubation with primary antibody, the sections were incubated with the secondary antibody and avidin-biotin-peroxidase complex. The slides were counterstained with H&E.

### Interpretation

#### HIF-1α

A positive value was recorded when nuclear staining was observed in >1% of cancer cells. Cytoplasmic staining was not counted. Sites of positive expression were also described in the following terms: superficial, deep-sited, diffuse, and vascular invasion.

#### P53

A positive value was recorded if distinct and strong nuclear staining was observed in >10% of the cancer cells.

#### VEFG

We evaluated the proportion (+1~+3) and intensity (+1~+3) of cytoplasmic staining of the tumor cells. We then multiplied the proportion value by the intensity value. After calculating the VEGF average value, underexpression of VEGF was defined below the average value; VEGF overexpression was defined as above average values. All evaluations for immunostaining were conducted by two independent observers with no knowledge of the patient's clinical status. A double-headed light microscope was utilized.

Each patient underwent gastrectomy, and D2 or more extended lymph node dissection was usually conducted. Tissue specimens were examined for the following characteristics: depth of tumor invasion, presence of lymph mode involvement, macroscopic and histological type, tumor size and lymphovascular invasion. The staging of gastric cancer and the clinicopathological factors utilized in this study were based on the sixth edition of the American Joint Committee on Cancer (AJCC) Cancer Staging Manual [9].

### Statistical analysis

The two-tailed χ^2 ^test was conducted to determine the significance of the difference between the covariates. sVEGF data are presented as median value (interquartile range), with nonparametric analyses being employed to assess differences. The Mann-Whitney U test were used to evaluate differences between groups. sVEGF cut-off value for survival analysis determined by ROC curve. Survival durations were calculated via the Kaplan-Meier method. The log-rank test was employed to compare cumulative survival in the patient groups. In all tests, *P *< 0.05 was the threshold of statistical significance. The SPSS software program (version 12.0; SPSS Inc., Chicago, IL) was utilized in the analyses.

## Results

### Patient characteristics

A total of 114 patients were included in the current analyses (Table 1). They comprised 67 males and 47 females, with a median age of 59 years (range, 28–84 years). Sixty seven patients (58.8%) evidenced tumor sizes of ≥ 4 cm. Tumor penetrated serosa or involved adjacent structures were observed in 37 patients (32.5%). Seventy three patients (64%) had LN metastasis. The postoperative stages of patients were I, II, III, and IV in 42, 25, 31, and 16 patients, respectively. There was no distant metastasis.

**Table 1:** Clinicopathological features

	No. of patients	%
Total number of patients	114	
Sex		
Male	67	58.8
Female	47	41.2
Age		
Median (Range)	59 (28–84)	
Lymphovascular invasion		
Negative	64	56.1
Positive	50	43.9
Lauren classification		
Intestinal	45	39.5
Diffuse	42	36.8
Mixed	18	15.8
Undetermined	9	7.9
Tumor size		
<4	47	41.2
≥4	67	58.8
T stage		
T0–2	77	67.5
T3–4	37	32.5
N stage		
N0	41	36.0
N1	46	40.4
N2	17	14.9
N3	10	8.8
TNM Stage		
I	42	36.8
II	25	21.9
III	31	27.2
IV	16	14.0

### Clinicopathological significance of HIF-1α, VEGF, and p53 protein expression

**Figure 1:**
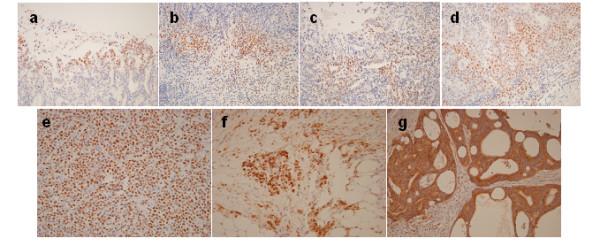
**Immunohistochemical staining of HIF-1α, p53, and VEGF**. Sites of positive expression of HIF-1α; superficial (a), deep-sited (b), diffuse (c), and vascular invasion (d). Cytoplasmic immunostaining of p53 (e). Underexpression (f) and overexpression (g) of VEGF. Original magnifications: ×200.

The relationships between HIF-1α, VEGF, p53 protein expression levels and clinicopathologic variables are provided in Figure 1 and Table 2. The positive rates of p53 and HIF-1α (diffuse, deep, intravascular pattern) were 38.6% and 15.8%, respectively. The VEGF overexpression rate was 57.9%. p53 and HIF-1α (diffuse, deep, intravascular pattern) were correlated positively with invasion depth (*P *= 0.015, *P *= 0.001, respectively). p53 was correlated significantly with LN involvement (*P *= 0.04). Correlations between VEGF overexpression and pathological status were not statically significant. However, VEGF overexpression was observed more frequently in the old age group (≥ 60 years old) and intestinal type (*P *= 0.013, *P *= 0.014, respectively).

**Table 2:** Correlation between the expression of HIF-1α, p53, VEGF, and preoperative sVEGF level and clinicopathological parameters

		**p53**			**HIF-1α**			**VEGF**			**sVEGF**	
		
		-	+	P*	-/S	D/V	P*	U	O	P*	Median (range)	P^†^
							
**Total**		70	44		96	18		48	66		(pg/ml)	
**Gender**												
Male	67 (58.8)	37	30	0.106	57	10	0.763	24	43	0.105	82 (6–1076)	0.275
Female	47 (41.2)	33	14		39	8		24	23		158 (7–642)	
**Age**												
<60	58 (50.9)	36	22	0.882	49	9	0.935	31	27	**0.013**	112 (6–622)	0.309
≥ 60	56 (49.1)	34	22		47	9		17	39		121 (6–1076)	
**Tumor size**												
<4 cm	47 (41.2)	34	13	**0.045**	43	4	0.061	19	18	0.761	76 (6–495)	**0.002**
≥4 cm	67 (58.8)	36	31		53	14		29	38		167 (6–1076)	
**T stage**												
T0,1	29 (25.5)	24	5	**0.015**	28	1	**0.001**	12	17	0.748	T0–2	0.565
T2	48 (42.1)	28	20		42	6		23	25		103 (6–1076)	
T3	31 (27.2)	14	17		23	8		10	21		T3–4	
T4	6 (5.3)	4	2		3	3		3	3		165 (8–504)	
**LN meta**												
Negative	41 (35.9)	30	11	**0.040**	38	3	0.051	17	24	0.917	77 (6–477)	**0.010**
Positive	73 (64.1)	40	33		58	15		31	42		156 (6–1076)	
**TNM stage**												
I	42 (36.8)	30	12	0.296	39	3	**0.022**	17	25	0.840	Stage I-II	0.382
II	25 (21.9)	15	10		21	4		14	11		96 (6–1076)	
III	31 (27.2)	13	18		25	6		7	24		Stage III-IV	
IV	16 (14.0)	12	4		11	5		10	6		158 (8–504)	
**Lauren**												
Intestinal	45 (51.7)	26	19	0.393	39	9	0.387	14	31	**0.014**	96 (6–1076)	0.294
Diffuse	42 (48.3)	28	14		37	5		24	18		154 (8–633)	

P * by χ^2 ^test, *P*^† ^by Mann-Whitney U test

HIF-1α: hypoxia-inducible factor 1 alpha, VEGF: vascular endothelial growth factor, sVEGF: preoperative serum vascular endothelial growth factor, LN: lymph node, -/s: negative/superficial pattern, d/v: deep/diffuse/vascular invasion pattern, U: under-expressed, O: over-expressed

### Clinicopathological significance of preoperative sVEGF levels

We noted a significant correlation between sVEGF levels and tumor size with higher sVEGF levels detected at the ≥ 4 cm tumor size (*P *= 0.002). Although the relationship between sVEGF levels and the invasion depth of the tumor was not statistically significant (*P *= 0.565), the presence of LN involvement was statistically significant (*P *= 0.010). Cut-off value of preoperative sVEGF for survival analysis was 105 pg/ml by ROC curve. 53.5% of patients had elevated preoperative sVEGF than cut-off value.

### Prognostic significance of preoperative sVEGF level, HIF-1α, VEGF, and p53 protein expression

**Figure 2:**
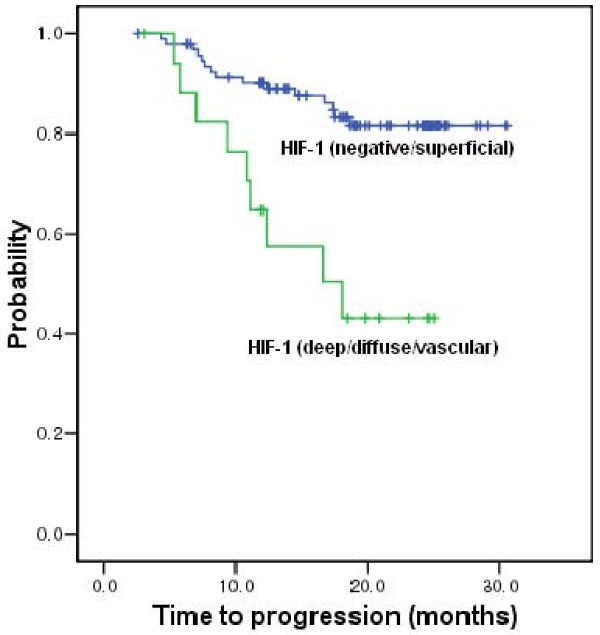
Time to progression curve according to HIF-1α expression (*P *= 0.002).

The median follow-up duration was 24.5 months (range, 14–32 months) after operation. HIF-1α (diffuse, deep, intravascular pattern) was observed to be a poor prognostic factor for disease recurrence or progression (*P *= 0.002) (Figure 2). However, we noted no significant association between the other factors and disease progression or patient overall survival.

### Association between HIF-1α, p53, and VEGF expression and preoperative sVEGF level

Although HIF-1α, p53, and VEGF expression and preoperative sVEGF level were correlated with several clinicopathologic findings, correlations between each factor were not observed (Table 3).

**Table 3:** Correlation of HIF-1α, p53, and VEGF expression with preoperative sVEGF level

	**p53**			**HIF-1α**			**VEGF**		
	
	-	+	*P*	-/S	D/V	*P*	Under	Over	*P*
**HIF-1α**									
-/S	58	38	0.617*						
D/V	12	6							
**VEGF**									
Under	33	15	0.169*	40	8	0.827*			
Over	37	29		56	10				

**sVEGF **(pg/ml)									
Median	103	139	0.705^†^	147	77	0.438^†^	153	163	0.146^†^
(range)	(8–1076)	(6–642)		(6–1076)	(6–761)		(8–1076)	(6–761)	

* by χ^2 ^test, ^† ^by Mann-Whitney U test

HIF-1α: hypoxia-inducible factor 1 alpha, VEGF: vascular endothelial growth factor, sVEGF: preoperative serum vascular endothelial growth factor, -/S: negative/superficial pattern, D/V: deep/diffuse/vascular invasion pattern, Under: under-expressed, Over: over-expressed

## Discussion

Tumor angiogenesis and neovascularization require VEGF expression and the binding of HIF-1α to the VEGF promoter is a major pathway resulting in the induction of VEGF expression under hypoxic conditions [10]. HIF-1α overexpression has been detected via IHC in several human cancers, including those of the brain, bladder, breast, colon, ovary, pancreas, kidney, and prostate [11-17]. Furthermore, HIF-1α overexpression is significantly correlated with highly aggressive disease and poor prognosis in some cancer types, including breast, ovarian, oligodendroglioma, and oropharyngeal cancers [13,14,18,19]. Our data also indicate that increasing HIF-1α expression performs an important function in tumor invasiveness and correlation with the TNM stage. In our study, HIF-1α expression grouping was different from that reported in previous studies. Because gastric cancer mass is not generally associated with tissue necrosis, like aggressive solid tumors (ex. glioblastoma multiforme) and HIF-1α is also expressed in benign ulcer lesions, particularly in the healing state [20], we classified the group of HIF-1α expression as a negative/superficial and deep/diffuse/vascular invasion pattern. Actually, in this study, superficial HIF-1α expressing patients' (limited within mucosa and submucosa) clinicopathological patterns were similar to those evidenced by HIF-1α negative patients.

In addition to the role of HIF-1α as a DNA-binding protein, HIF-1α has also been shown to exert biological effects via protein-protein interactions. Under hypoxic conditions, HIF-1α has been shown to interact with the tumor suppressor protein p53, which itself is a DNA-binding force for p53 loss of function in gastric cancer [7]. The prognostic value of p53 remains controversial, as the majority of previous studies have reported an association of p53 with patient survival [21], while some other investigations contradict these findings [22]. In our study, p53 expression was correlated with tumor size, tumor invasion, and LN metastasis. However, we noted no association between the expressions of HIF-1α and VEGF. Survival prognostic predictivity was also not observed.

Tumor VEGF expression was shown to be a significant marker for tumor recurrence or reduced survival independent of conventional clinicopathological variables in several cancers [23]. In cases of gastric cancer, a positive correlation between VEGF expression and lymphatic invasion, LN metastasis, venous invasion, and patient outcome has been reported by several groups [21,24,25], but there are also published reports that contradict this notion [26,27]. In our observations, VEGF overexpression is not correlated with clinopathological findings except in the elderly and in cases of intestinal-type gastric cancer. Although we evaluated cytoplasmic VEGF expression including proportion and intensity, the results remained the same.

Higher preoperative sVEGF levels have been reported in gastric cancer patients as compared with healthy controls, and this has been clinically correlated with stage, and the presence of distant metastasis [28-31]. In our study, tumor size and LN metastasis were correlated with sVEGF levels.

HIF-1α, p53, and VEGF expression have been reported to be positively correlated. However, correlations between preoperative sVEGF level and tissue HIF-1α, VEGF, and p53 were not observed in this study. These results, which differ from those of other studies, may be attributable to differences in the methods of the studies, including the criteria of positivity and the number of patients studied. Additionally, sVEGF is regulated by several potential transcription factor-binding sites, including HIF-1α, activator protein 1, activator protein 2, Egr-1, Sp1, and a host of others. Furthermore, angiogenesis is influenced by several endogenous pro-angiogenic factors and anti-angiogenic factors [10]. Further studies will be required in order to determine the definitive mechanisms inherent to metastatic pathogenesis, thus allowing us to develop more effective cancer treatments.

## Conclusion

Our data also indicate that p53, HIF-1α, and preoperative sVEGF could be markers of invasion depth or LN involvement. Although the median follow-up duration was too short to analyze, HIF-1α expression was found to be a poor prognostic factor for disease recurrence or progression in patients with gastric cancer.

## Competing interests

The authors declare that they have no competing interests.

## Authors' contributions

SYO carried out data analysis and had written the manuscript. H–CK designed this study protocol and participated in manuscript review. S–HK participated in data collection and drafted manuscript. JSJ participated in data collection. MCK carried out operation and data collection. KHK and J–YH carried out sVEGF immunoassay. S–JK, J–SJ and COK carried out immunohistochemical staining and interpretation. H–JK participated in study coordination and correspondence.

## Pre-publication history

The pre-publication history for this paper can be accessed here:


